# Retrogasserian Glycerol Rhizotomy (RGGR) for Trigeminal Neuralgia Induced by Postvaccination Herpetic Reactivation: A Rare Case Report

**DOI:** 10.1002/ccr3.70814

**Published:** 2025-08-22

**Authors:** M. Kodeeswaran, M. Naveen Kumar, K. P. Priyadharshan, Arun Narinder, M. Panchabakesan, B. C. Suresh Kumar, Bipin Chaurasia

**Affiliations:** ^1^ Neurosurgery Academy and Research Foundation, Department of Neurosurgery Government Kilpauk Medical College and Hospital Chennai Tamil Nadu India; ^2^ Department of Neurosurgery Government Kilpauk Medical College and Hospital Chennai Tamil Nadu India; ^3^ Apollo First Med Chennai Tamil Nadu India; ^4^ Department of Neurosurgery College of Medical Sciences Bharatpur Nepal

**Keywords:** COVID vaccination, herpes zoster, percutaneous retrogasserian glycerol rhizotomy, trigeminal nerve, trigeminal neuralgia

## Abstract

Reports on surgical treatment for postherpetic trigeminal neuralgia after COVID vaccination have not been found in the literature. Here, we described a case of postherpetic trigeminal neuralgia after COVID vaccination that was treated with percutaneous retrogasserian glycerol rhizotomy (PRGR) resulting in complete pain relief. We reported a case involving a 70‐year‐old female who had herpes zoster infection in the ophthalmic branch (V1) dermatome following COVID vaccination and presented with paroxysmal electric shock‐like sensation without a trigger. Her pain was refractory to medical management. Her Barrow Neurological Institute pain intensity score was IV. Magnetic resonance imaging of the brain was normal. PRGR was performed. The patient's pain decreased over the next 2 h, and she was pain‐free from post‐procedure Day 2 while taking carbamazepine 400 mg/day, which was tapered over 2 weeks. At her 3‐month follow‐up, the patient was reviewed and remained pain‐free. Percutaneous treatment of postherpetic trigeminal neuralgia may be considered the treatment of choice in vaccination‐induced postherpetic trigeminal neuralgia.

**Summary**

- In the aftermath of COVID‐19 vaccination, the emergence of trigeminal neuralgia is identified as a seldom observed occurrence. Given the absence of any vascular loop surrounding the trigeminal nerve, we advocate for the implementation of percutaneous retrogasserian glycerol rhizotomy (PRGR) as the primary intervention for this unusual clinical presentation.

## 1 Introduction

Postherpetic trigeminal neuralgia (TN) is characterized by unilateral facial pain that persists for at least 3 months after herpes zoster infection and is generally expected to improve within 6 months to 1 year. However, managing this condition can be challenging, leading to chronic pain and substantially reducing the quality of life (QOL) [1, 2]. Treatment for postherpetic TN is mainly conservative, although there have been a few reports of surgical treatments, such as radiofrequency ablation, percutaneous balloon compression, and microvascular decompression [3, 4]. We present a case of a TN patient who had herpes zoster infection in the right trigeminal nerve, which was activated post–COVID vaccination. The patient underwent percutaneous retrogasserian glycerol rhizotomy (PRGR) with positive results.

## 2 Case History

**FIGURE 1:**
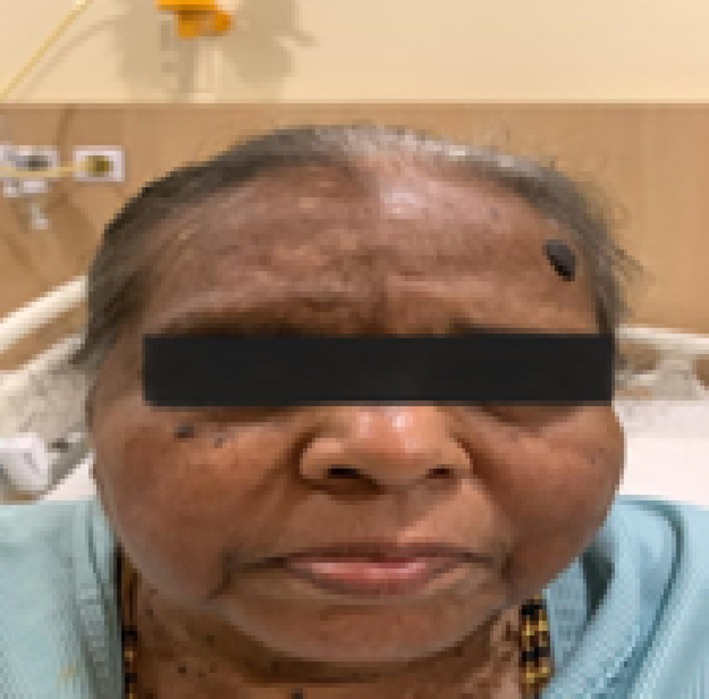
Healed vesicles along the V1 distribution.

A 70‐year‐old female presented with right‐sided facial pain for 2 years, which she described as an electric shock‐like sensation along her right eye, accompanied by burning pain and numbness in the right trigeminal nerve dermatome. Two years prior, she had received a COVID vaccination. Following the vaccination, she was diagnosed with a herpes zoster infection characterized by persistent right facial pain occurring in the first branch of the right trigeminal nerve. She was prescribed several medications, but her condition became refractory to treatment. Her medications were no longer effective, so she anticipated undergoing other possible treatments. No family history was observed. Her Barrow Neurological Institute (BNI) pain intensity score was grade IV (Table 1). The patient experienced facial pain that severely limited her daily activities. She exhibited healed vesicles along the V1 distribution (Figure 1). No other obvious symptoms of neurological deficits were observed. Cranial nerve examination was normal. There were no focal neurological deficits.

**TABLE 1:** BNI pain intensity score [5].

BNI score features	
1	No pain, no medications
2	Occasional pain, no medications required
3a	No pain, continues to use medications
3b	Some pain, adequately controlled with medications
4	Some pain, not adequately controlled with medications
5	Severe pain, no relief of pain

Abbreviation: BNI, Barrow Neurological Institute.

## 3 Methods

**FIGURE 2:**
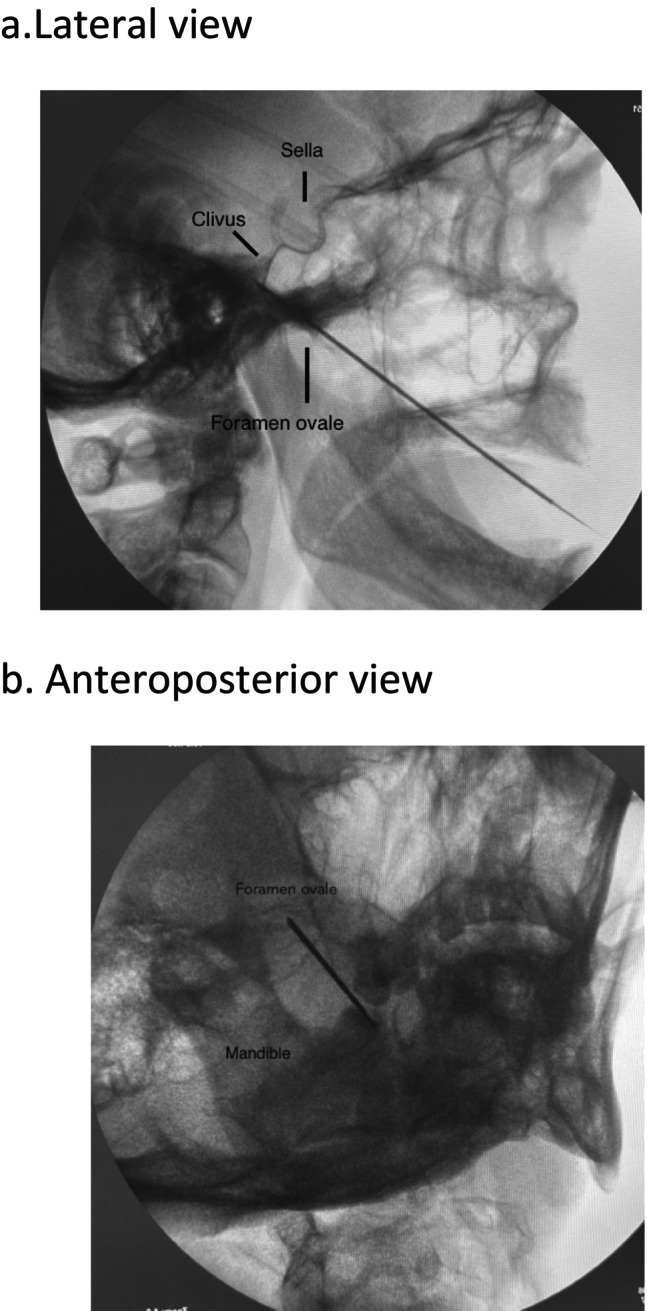
The procedure was performed under fluoroscopic guidance.

Magnetic resonance imaging (MRI) suggestive of no vascular loop showed no neoplastic lesions in the right cerebellopontine angle. At our center, PRGR is offered as first‐line therapy in patients with idiopathic TN. PRGR was performed on this patient, who was continuously monitored in the operating room for blood pressure, heart rate, oxygen saturation, and respiratory function. Adequate sedation for the patient and a quick response to any cardiovascular changes that might occur during the procedure were essentially performed. Most of the discomfort experienced during PRGR occurs when the 18‐gauge spinal needle was passed through the foramen ovale. The procedure is begun with the patient supine position but completed in the sitting position with neck flexed during glycerol injection, a balance of pain control, blood pressure management, and respiratory care must be maintained by the surgeon and anesthesiologist [6]. Under C‐arm guided fluoroscopic image, 18‐gauge needle was inserted and around 2 mL of glycerol was injected. The whole procedure was performed under fluoroscopic guidance (Figure 2a,b). Post‐procedure, the patient was placed into a sitting position with flexion of the neck.

## 4 Results

Patient's pain reduced over the next 2 h of post‐procedure and patient was pain‐free from Day 2, on carbamazepine 400 mg/day, which was tapered over 2 weeks. The patient was reviewed at her 3‐month follow‐up and remained pain‐free.

## 5 Conclusion

Post–COVID vaccination–induced TN is a rare condition. In the absence of a vascular loop around the trigeminal nerve, we cautiously recommend PRGR as a first‐line procedure for this unusual clinical presentation. However, due to the rarity of this condition, it is crucial to carefully assess each case before proceeding with this intervention.

## 6 Discussion

Recurrent episodes of hemifacial pain are a hallmark of postherpetic TN. In the acute stage, the individual experiences sensory anomalies that vary from nociceptive discomfort to neuropathic discomfort. These pathological conditions are attributed to abnormalities within the trigeminal nerve, the gasserian ganglion, or the root entry zone [7]. Population analyses show a TN prevalence of 52.4% in rural settings compared to 47.6% in urban settings. In terms of frequency, the left side of the face (38.8%) is affected less often than the right side (57.1%). Studies show that the occurrence of TN is approximately 2–5 cases for every 100,000 individuals per year, with a noticeable increase with advancing age. In particular, among those aged 60–69 years, the incidence increases to 17.5 for every 100,000 annually, and it further climbs to 25.6 per 100,000 each year once individuals reach 70 years of age.

Jannetta et al. [8] have attributed the etiology of TN to compression of the trigeminal nerve by arterial loops or veins. Several studies, including that of Ramesh and Premkumar [9], have shown that the vascular contact and compression at the trigeminal root entry zone can also be seen in a significant portion of the normal population. However, in our case, the patient's imaging did not reveal any vascular loop. The treatment of choice for refractory TN was not properly determined due to a lack of knowledge in the etiopathogenesis of TN. Various procedures, such as balloon micro compression, radiofrequency rhizotomy, glycerol rhizotomy, and stereotactic radiosurgery, have been followed to induce axonal degeneration of the nerve [9]. While these procedures can provide excellent pain relief, they are also associated with recurrences in the late follow‐up period. Kondziolka and Lunsford (2005) reported recurrence in about 23% of 1174 cases after PRGR [10]. Kodeeswaran et al. (2015) reported a recurrence rate of about 11% after PRGR on long‐term follow up [5].

The recurrent TN addressed through the application of PRGR, as documented by Kodeeswaran et al. in 2019, encompassed a total of 11 patients in their investigation who experienced recurrence following various therapeutic interventions, all of whom were subsequently managed with PRGR. The evaluations performed following the procedure revealed that there were no signs of recurrence in these patients. Therefore, the determination of the procedural technique depends on the patient's symptomatology, existing health issues, age, and previously employed treatment methods. The primary objective of the intervention should be to achieve comprehensive pain alleviation while maintaining an acceptable incidence of adverse effects. It is imperative that the patient experiences an absence of pain and diminished anxiety regarding potential recurrence.

Pollock reported in his series of 98 patients that 73% were free of pain following the surgical procedures. The probability of these patients remaining pain‐free without medications after 1 and 3 years was 61% and 50%, respectively. His study shows that the reported outcomes are almost always comparable across nearly all forms of treatment [11].

PRGR shows lesser sensory deficits than other percutaneous procedures; hence, it is considered the first line of treatment for refractory TN. Anhydrous glycerol chemically ablates the so‐called pathological site on the axons.

There are various procedures for the treatment of postherpetic TN (Table 2). However, the percutaneous method has not been proven superior to other surgical modalities. The most common complications of PRGR are minor, such as the development of a local hematoma, infection, sensory deficits, reactivation of labial herpes, and anesthesia dolorosa. Chemical meningitis and infectious meningitis are very severe complications of PRGR, but they are very rare. This is a relatively safe procedure and is well‐tolerated by patients of all age groups. It can be performed under local anesthesia with X‐ray or C‐arm guidance. Mild sedation may also be administered during the procedure if needed. Patients can typically return to their normal routine within a few days.

**TABLE 2:** Various studies involves postherpetic neuralgia [12, 13, 14, 15, 16].

Study	Year	Type of procedure	Pain reduction
Onoda Keisuke et al. [12]	2023	MVD	YES
Javier et al. [13]	2022	Pulsed RF	YES (side effects+)
Li Hongxi et al. [14]	2021	High RF	YES
Durgosik et al. [15]	2000	GAMMA KNIFE	YES
Kikuchi et al. [16]	1999	Intra thecal MP	50% Reduction

In this case report, the patient with TN experienced a distinct variant of postherpetic neuralgia (PHN) following the administration of the COVID vaccine. Initially, she developed the characteristic burning pain associated with PHN following COVID vaccination. Later, she developed the sudden electric shock‐like pain more typical of TN. There seem to be no other reported cases of patients experiencing these distinct consecutive conditions. Alternatively, one could hypothesize that the irritated or damaged sensory nerve from the PHN may have been predisposed to the microvascular insult classically associated with TN. Regardless of the mechanism, this novel case reminds the surgeons to be cognizant of the nuances and potential overlap of postvaccination‐induced herpetic and TNs without any pathophysiological links between the diseases.

## Author Contributions

**M. Kodeeswaran:** conceptualization, data curation, formal analysis, project administration, resources, supervision, validation, visualization, writing – review and editing. **M. Naveen Kumar:** conceptualization, data curation, formal analysis, investigation, methodology, visualization, writing – original draft, writing – review and editing. **K. P. Priyadharshan:** project administration, supervision, writing – review and editing. **Arun Narinder:** project administration, supervision, writing – review and editing. **M. Panchabakesan:** supervision, writing – review and editing. **B. C. Suresh Kumar:** project administration, supervision, writing – review and editing. **Bipin Chaurasia:** project administration, resources, supervision, writing – review and editing.

## Ethics Statement

The authors have nothing to report.

## Consent

Written informed consent was obtained from the patient.

## Conflicts of Interest

The authors declare no conflicts of interest.

## Data Availability

Data sharing not applicable—no new data generated, or the article describes entirely theoretical research.
